# Study on the dynamic response and roadways stability during mining under the disturbance of hard roof break

**DOI:** 10.1038/s41598-024-66376-4

**Published:** 2024-07-03

**Authors:** Kong Peng, Liu Chang, Yang Dechuan, Li Shihui, Jin Ruiju

**Affiliations:** 1 Engineering Laboratory for Safe and Precise Coal Mining of Anhui Province, Anhui University of Science and Technology, Huainan, 232000 China; 2 School of Mining Engineering, Anhui University of Science and Technology, Huainan, China; 3 Huaihe Energy Group, Huainan, China

**Keywords:** Hard roof, Stability of roadway, Dynamic response, Numerical simulation, Natural hazards, Coal

## Abstract

Under the condition that the working face was directly covered with hard roof, the abrupt breaking of hard roof release significant amount of energy, thus prone to triggering dynamic disasters such as roadway instability or rockburst. This paper based on the engineering background of the Xieqiao Coal Mine's 11,618 working face, a numerical simulation method was put forward to study the dynamic response of roadway under the disturbance of hard roof breaking and proposed an evaluation index *I*_*C*_ for roadway stability. Research indicates that the elastic energy released during the periodic weighting of the hard roof is higher than that released during the first weighting. Under the dynamic disturbance caused by hard roof breaking, the peak stresses of the roadway was slight decreased, accompanied by a significant increase in the range of stress concentration and plastic zone expansion. Roadway deformation patterns are significantly influenced by hard roof breaking, with noticeable increases in deformation on the roof and right side. During the period of hard roof breaking, the possibility of instability of the roadway increase significantly due to the disturbance caused by the dynamic load. The research results reveal the instability mechanism of roadway under the condition of hard roof, and provide a more reliable basis for evaluating the stability of roadway.

## Introduction

With the continuous increase of coal demand, the geological conditions of coal mining have become increasingly complex. Frequent occurrences of mining-induced dynamic disasters are attributed to the presence of hard roof. Statistics show that over 50% of coal seams in China's major coal-producing regions, such as Inner Mongolia, Shanxi Binchang, Shanxi Datong, Anhui Huainan, Henan Yima, Northeast China, and Shandong, are associated with hard roof composed mainly of sandstone and rudstone^1–3^. Under the conditions of hard roof occurrence, it is easy to form a large area of suspended roof after coal seam mining, which can lead to sudden roof collapse, triggering dynamic disasters such as rockburst, roadways instability, and coal and gas outbursts^4–6^.

The main cause of triggering dynamic disasters is the bending elasticity and sudden breaking induced dynamic disturbances generated by the large-scale overhanging of the hard roof^7,8^. Fu et al.^9^ employed theoretical analysis to establish a model of roof structure, revealing that as the thickness and strength of the hard roof increase, the cantilever length of the overlying roof in goaf also increases. Yang et al.^10^ and Zuo et al.^11^ analyzed the stress distribution pattern of the roof based on the medium-thick plate theory, proposing the break model of the hard roof and its mechanical criteria. Ma et al.^12^ established a predictive model for the energy of hard roof fracture motion based on the critical layer theory, and analyzed the additional perturbation stress caused by seismic waves on the working face. Wu et al.^13^ investigated the movement law of roof under the conditions of hard roof existence using a similar material simulation method. Experimental results showed that the step distance of hard roof breaking reached 270 m, indicating that the breaking of hard roof played a controlling role in the fractures and overlying strata structure.

Statistics indicate that over 80% of dynamic disasters such as rockburst occurred in the roadway. Tai et al.^14^ research found that after the hard roof broke, the rotating compression effect created by the arc-shaped triangular plates on both sides of the goaf leads to heightened stress in the coal pillars. This stress can result in deformation and damage to the roadway. Song et al.^15^ investigated that the relationship between roadway layout, synclinal structure, and rockburst accidents in the Xinjulong Coal Mine based on Bayesian full waveform inversion and simulation. The research showed that when the distance between the roadway and the seismic source was less than 60 m, the stability of the roadway was controlled by the intensity of the seismic source, whereas when the distance exceeded to 60 m, the stability of the roadway was controlled by the synclinal structure. Dai et al.^16^ employed stability theory, integrated dynamic disturbance factors such as geostress, mechanical properties of coal and rock, support strength, and roadway dimensions to establish a mechanical model of roadways under dynamic disturbance, obtained key indicators for roadway rock failure and rockburst occurrence. Ling et al.^17^ utilized a self-developed true triaxial rockburst test system to study the failure process of circular roadway under dynamic disturbance loads, analyzed roadway fracture mechanisms, acoustic emission characteristics, and fragment features. Kong et al.^18^, Han et al.^19^ used FLAC3D numerical simulation to investigate the deformation and instability laws of roadway under the combined action of dynamic and static loads. The research showed that the vibration velocity of the roof was significantly higher than that of the floor, and even with relatively small dynamic disturbances, significant increase in the plastic zone of roadway surrounding rock and deformation was observed, especially at greater burial depths.

Under the condition of hard roof occurrence, the static stress concentration degree of surrounding rock in stope is higher. In addition, the breaking of hard roof causes severe dynamic load disturbance to the surrounding rock, and the superposition of high static stress and strong dynamic load is easy to induce roadway instability. Therefore, in the study of roadway stability, we should not only analyze the static load stress of roadway surrounding rock, but also pay attention to the influence of dynamic load disturbance of hard roof breaking. However, during the mining process of the working face, the stress distribution of the surrounding rock of the roadway is complex, and there is currently a lack of definitive research on the dynamic response of the roadway under the dynamic disturbance of the hard roof breaking. In order to reveal the mechanism of dynamic instability induced by hard roof conditions, so as to evaluate the stability of roadway more accurately, further research is needed on the dynamic response law of roadway under the disturbance of hard roof breaking. This paper takes the 11,618(east) working face of Xieqiao Coal Mine as the engineering background and adopt theoretical analysis methods to study the breaking law of hard roof under direct overlay conditions. Based on FLAC3D numerical simulation software, a numerical simulation method for calculating the dynamic effects of hard roof breaking is proposed. The characteristics of multi-field dynamic response of surrounding rock in roadway under dynamic disturbances caused by roof breaking are analyzed, and stability evaluation indicator for roadway under dynamic disturbance is proposed. The research results provide a theoretical basis for controlling the stability of roadways under the condition of hard roof.

## Engineering background

**Figure 1:**
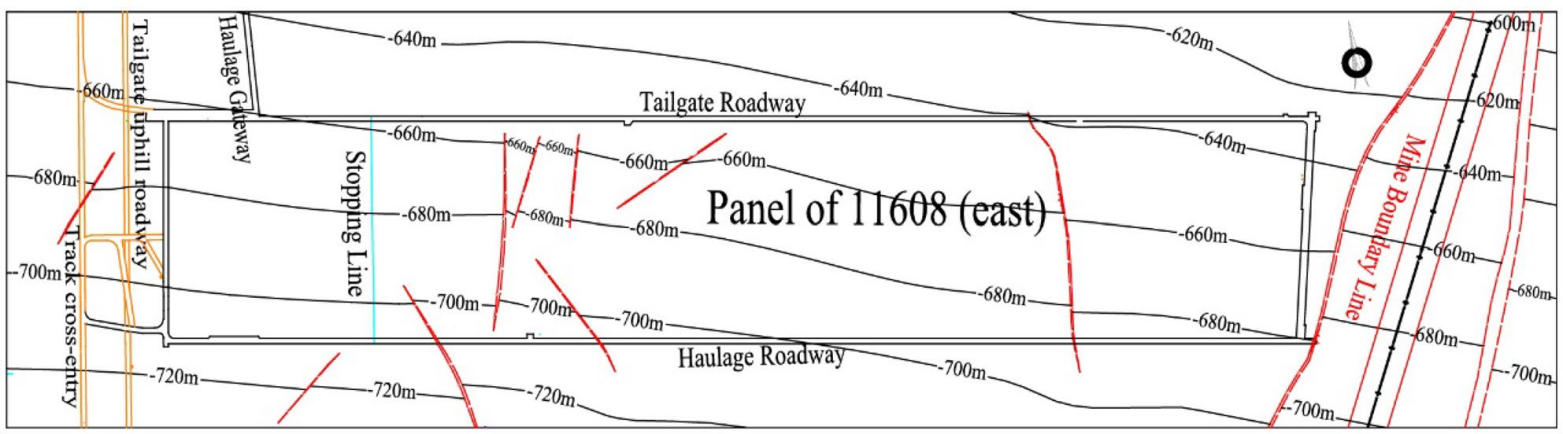
Panel of 11,618(east) mining engineering plan.

The 11,618(east) working face of Xieqiao Coal Mine with a width of 230 m and a length of 1000 m. The working face adopts the longwall mining method along the strike, with roof caving method used to handle the goaf. The thickness of the coal seam ranges from 0 to 4.8 m and the average thickness is 3.1 m. The layout of the working face is shown in Fig. 1.

**Figure 2:**
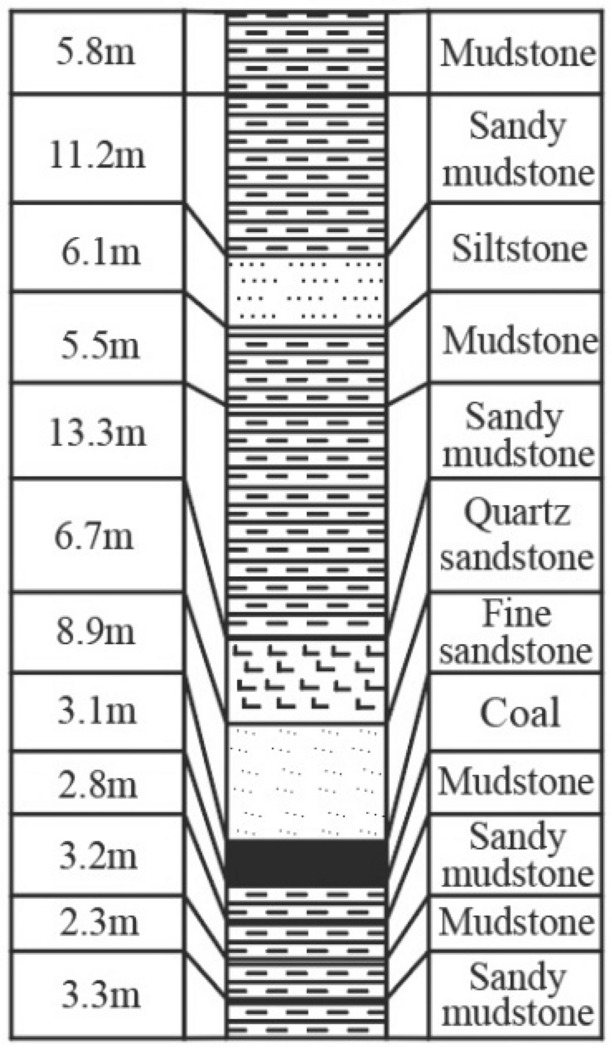
The histogram of Panel 11,618 (east).

The thickness of the fine sandstone ranges from 4.4 to 16.7 m, with an average thickness of 8.9 m, most of it directly covers above the coal seam. The uniaxial compressive strength of the fine sandstone reaches 180 MPa, therefore, the 11,618 (east) working face belongs to a typical hard roof directly overlying working face. Above the fine sandstone layer is quartz sandstone, with an average thickness of 6.7 m and the uniaxial compressive strength is 89 MPa. The rock layer within 40 m above quartz sandstone is mainly composed of siltstone, mudstone, and sandy mudstone, with uniaxial compressive strength not exceeding 70 MPa. The strength and integrity of these rock formations are significantly lower compared to the fine sandstone and quartz sandstone layers. The immediate floor is mudstone, with a thickness ranging from 0.5 to 3.4 m and an average thickness of 2.1 m, while the main floor is siltstone with a thickness of 1.2–6.0 m and an average thickness of 3.6 m. The histogram of the roof and floor is depicted in Fig. 2.

## The regular pattern of hard roof breaking and energy release

### Analysis of hard roof breaking

**Figure 3:**
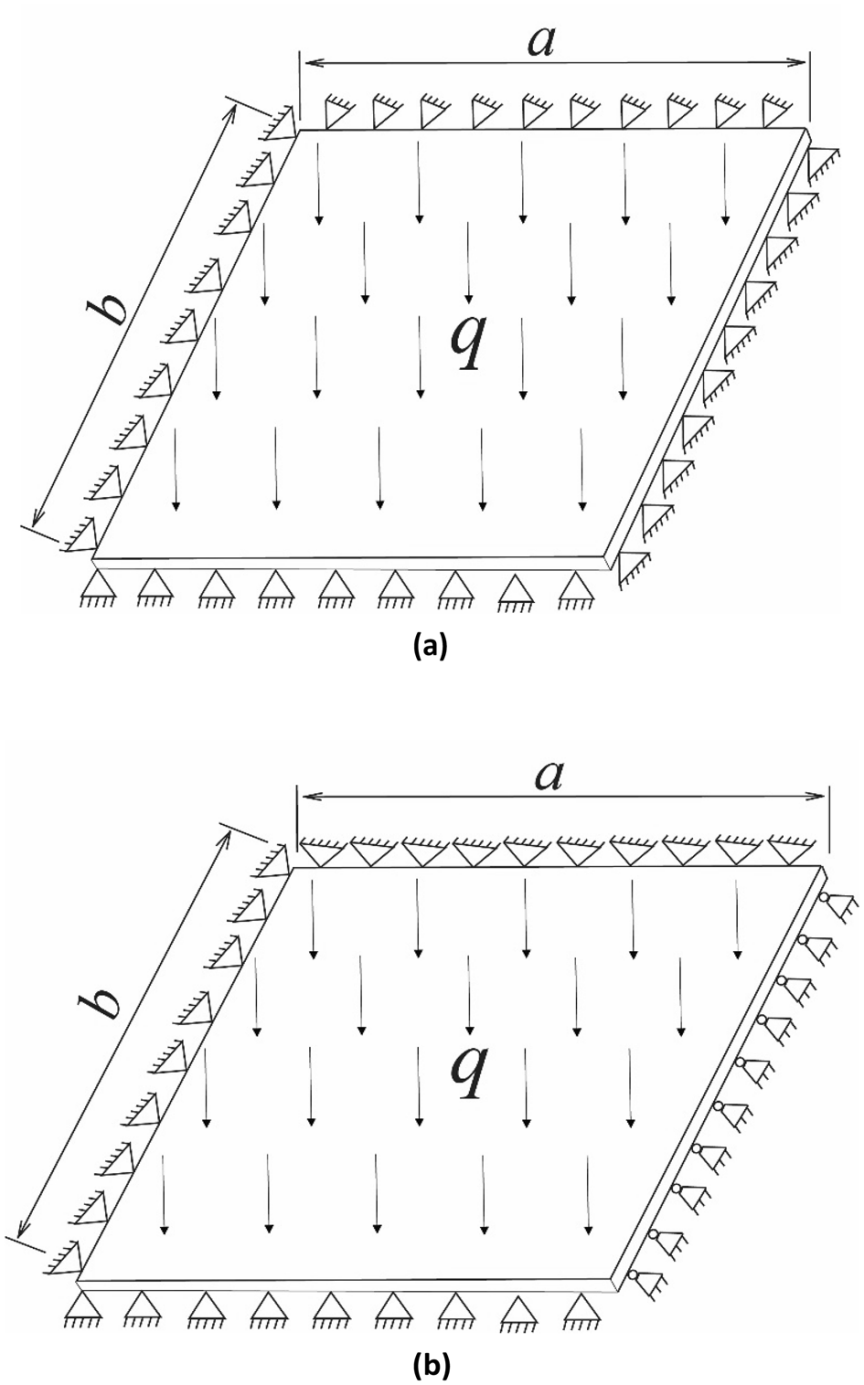
Boundary conditions of the fine sandstone roof (**a**) First weighting (**b**) Periodic weighting.

The large breaking step distance of the hard fine sandstone directly overlying the working face plays a controlling role in the evolution of the roof structure. Based on thin plate theory and mining pressure theory^20–22^, the boundary conditions of the fine sandstone roof during the initial mining period of the working face are considered as fixed support on all four sides. Therefore, the formula for the hard roof breaking step during the first weighting can be calculated as shown in Eq. (1). After the first weighting, the boundary conditions of the roof on the goaf side can be considered as simply supported. Hence, during the periodic weighting of the hard roof, the boundary conditions of the roof are three sides fixed support and one side simply supported, and the roof breaking step distance can be calculated using Eq. (2). The boundary conditions of the roof during the first and periodic weightings are illustrated in Fig. 3.

Under the condition of fixed support on all four sides, the breaking step distance of hard roof is:

1 $${a_1} = \left\{ \begin{array}{ll} {b \cdot \sqrt[4]{{l_m^2}/({b^2} - l_m^2)},}&\left( {{l_m} < b <\sqrt 2 {l_m}} \right) \\ \frac{b}{{\sqrt 2 {l_m}}} \cdot \sqrt {{b^2} - \sqrt {{b^4} - 4l_m^4} } ,&\left( {b \geq \sqrt 2 {l_m}} \right) \end{array} \right.$$

Under the condition of three sides fixed and one side simply supported, the breaking step distance of hard roof is:

2 $${a_2} = \left\{ {\begin{array}{*{20}{l}} {\sqrt {{\text{b}} \cdot \sqrt[{4}]{{\frac{{{\text{2l}}_{\text{m}}^{2}}}{{{2(}{b^2} - l_m^2{)}}}}}} ,}&{\left( {{l_m} < b < \sqrt[{4}]{{2}}{l_m}} \right)} \\ {\frac{b}{{l_m}} \cdot \sqrt {{b^2} - \sqrt {{b^4} - {2}l_m^4} } ,}&{\left( {b \geq \sqrt[{4}]{{2}}{l_m}} \right)} \end{array}} \right.$$

The stepping index *l*_m_ in formulas 1 and 2 can be expressed as:

3 $${l}_{m}=\frac{h}{1-{\mu }^{2}}\sqrt{\frac{2{\sigma }_{s}}{q}}$$

In the equation, *u*—the Poisson's ratio of the rock formation; *q*—The self-weight and applied loads on the rock strata, N/m^2^; *a*_1_, *a*_2_—The advancing distance of the working face, m; *b*—The length of the working face, m; *h*—The thickness of the rock strata, m; *σ*_*s*_—Tensile strength of the rock strata, Mpa.

According to the test results of rock mechanics parameters, the Poisson's ratio *u* of fine sandstone is 0.28, and the tensile strength *σ*_*s*_ is 11.65 MPa. The self-weight and applied loads on the rock strata *q* are calculated based on a load layer thickness 15 times the mining height, with a value of 1.16 MPa. The working face length *b* is taken as 230 m, and the thickness of rock strata *h* is taken as 8.9 m. Firstly, by substituting the above parameters into Formula 1, the stepping index *l*_*m*_ is calculated as 43.28 m. Then, Formulae 1 and 2 are sequentially calculated, indicating that: under the condition of fixed support on all four sides, in other words, during the first weighting of fine sandstone, the breaking step distance of the roof is 43.31 m; under the condition of three sides fixed and one side simply supported, in other words, during the period weighting of fine sandstone, the breaking step distance of the roof is 43.29 m. Due to the large working face length of 230 m, the breaking step distance of the roof under condition of three sides fixed and one side simply supported is slightly reduced compared to that of fixed support on all four sides, indicating a slight decrease of the periodic weighting step distance compared to the first weighting step distance.

### The regular pattern of energy release during roof fracturing

The elastic energy released during the breaking of hard roof is manifested in the form of mine tremors, propagating energy to the surrounding rock, thereby inducing dynamic disturbances in the surrounding rock of the mining area, which can lead to instability of roadways. Considering the roof fine sandstone as a beam model, during the first fracturing, thus the elastic energy released during the first fracturing of fine sandstone can be represented by Eq. 4. When the roof fine sandstone undergoes the periodic fracturing, the formula for the elastic energy released during the periodic weighting of the fine sandstone is represented by Eq. 5^23,24^.

4 $${U}_{fw}=\frac{\sqrt{2}{l}_{fw}{h}^{2}{R}_{t}^{2.5}}{24\sqrt{q}E}$$

5 $${U}_{pw}=\frac{{l}_{pw}{h}^{2}{R}_{t}^{2.5}}{6\sqrt{3}\sqrt{q}E}$$

In the equation: *U*_*fw*_ represents the elastic energy released during the first weighting of the main roof; *U*_*fw*_ represents the elastic energy released during the periodic weighting of the main roof; *l*_*fw*_ represents the first weighting step of the main roof; *l*_*pw*_ represents the periodic weighting step of the main roof; *q* represents the load per unit length, converted from the self-weight of the main roof and the additional loads imposed by the overlying strata; *E* represents the elastic modulus of the main roof; *R*_*t*_ represents the tensile strength of the main roof; and *h* represents the thickness of the main roof.

**Figure 4:**
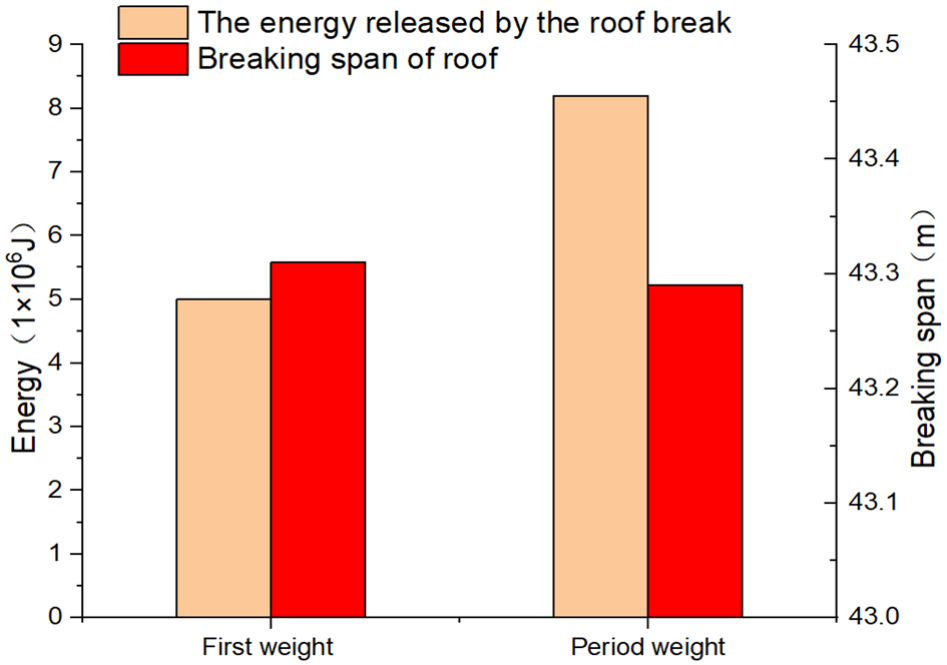
Breaking step distance and energy release of the hard roof.

Based on the actual conditions of the 11,618 working face, the load per unit length converted from the self-weight of the main roof and the additional loads imposed by the overlying strata q is taken as 1.16 MPa. The elastic modulus *E* of the main roof is assumed as 17.3 GPa, the tensile strength Rt of the main roof is considered as 11.65 MPa, and the thickness h of the main roof is 8.9 m. The unsupported spans for the first weighting *l*_*fw*_ and periodic weighting *l*_*pw*_ of the main roof are determined as 43.31 m and 43.29 m, respectively. Substituting these parameters into Formulae 4 and 5, the elastic energies released during first weighting and periodic weighting are calculated as 5.0 × 10^6^ J and 8.2 × 10^6^ J. The first and periodic breaking span of the hardroof, as well as the energy released, are shown in Fig. 4.

## Simulation methodology

### Establishment of modeling

When the hard roof is first broken and periodically broken, a large amount of energy is released, and the energy propagates in the form of dynamic stress waves from the source to the surrounding areas. As the dynamic stress waves propagate to the advanced roadway area of the working face, there will be significant changes in the stress, plastic zone development, vibration velocity, deformation of the roadway. However, the stress distribution and dynamic stress wave propagation laws during the mining are extremely complex, posing significant challenges to the study of the dynamic response of mining fields. The FLAC3D numerical simulation can accurately reproduce the three-dimensional static stress distribution characteristics of the mining area, and combined with the dynamic analysis module, simulate the dynamic load released by the breaking of the hard roof. The dynamic response of the roadway under the action of hard roof breaking disturbance is studied, which provides a basis for the prediction and prevention of dynamic disasters under the condition of hard roof.

**Figure 5:**
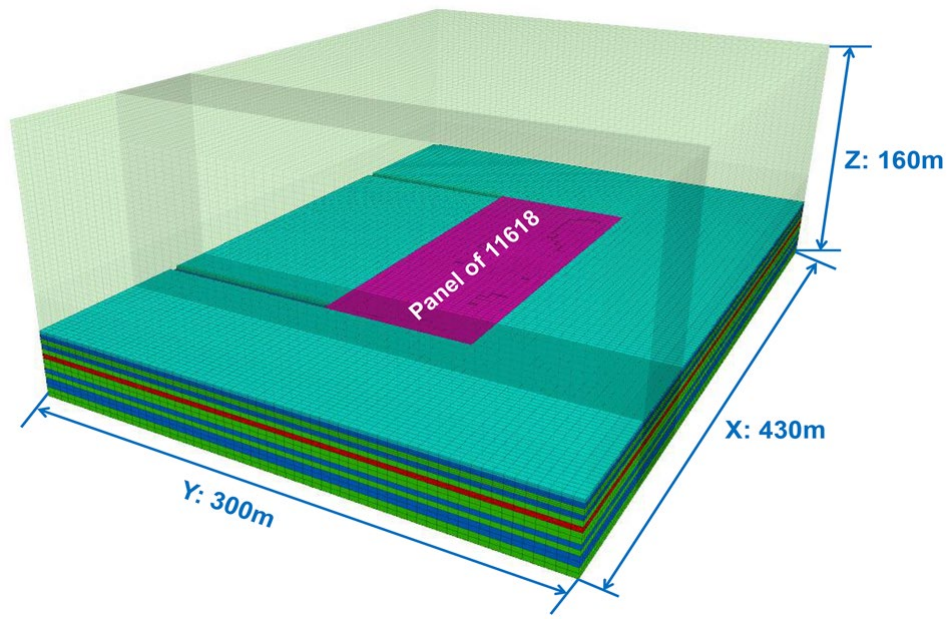
Numerical computational model.

Taking the 11,618(east) working face of Xieqiao Coal Mine as the engineering background, a large scale three-dimensional numerical computational model is established. The model dimensions are 430 m in length (X), 300 m in width (Y), and 160 m in height (Z), comprising a total of 1,093,500 grids and 1,131,184 nodes. The numerical computational model is illustrated in Fig. 5. The boundaries of the model are fixed, and the simulated working face is buried at a depth of 700 m. The initial geostress are applied to the model boundary based on on-site stress testing results, with lateral pressure coefficients of 0.6 in the X direction and 0.8 in the Y direction.

Excavation of the working face causes redistribution of stress in the surrounding rock, exhibiting strain-softening characteristics after damage and failure occur in the rock mass. Therefore, in the numerical computational model, the constitutive model for the coal and rock mass is chosen as the Mohr–Coulomb strain-softening model. The parameters of the coal and rock mass are shown in Table 1.

**Table 1:** Coal and rock mass properties.

Lithology	*E* (GPa)	*υ*	*C* (MPa)	*σ* (MPa)	*φ* (deg.)	*c*_*r*_ (MPa)	*ε*_*p*_ (%)
Fine sandstone	17.3	0.28	8.6	7.3	48	0.86	0.01
Quartz sandstone	14.0	0.29	6.1	5.4	45	0.61	0.01
Mudstone	6.9	0.31	2.8	2.4	38	0.31	0.01
Sandy mudstone	8.1	0.30	3.2	2.7	40	0.30	0.01
Coal	2.4	0.34	1.8	1.3	36	0.18	0.01
Siltstone	12.7	0.27	5.4	4.8	43	0.54	0.01

*E*_*m*_ is the elastic modulus, *μ* Poisson's ratio, *C* is cohesive, *σ* is tensile strength, *φ* is the internal friction angle, and *c*_*r*_ is the residual cohesive, *ε*_*P*_ is the plastic strain when the strength of the rock mass becomes residual.

### Dynamic analysis procedures

**Figure 6:**
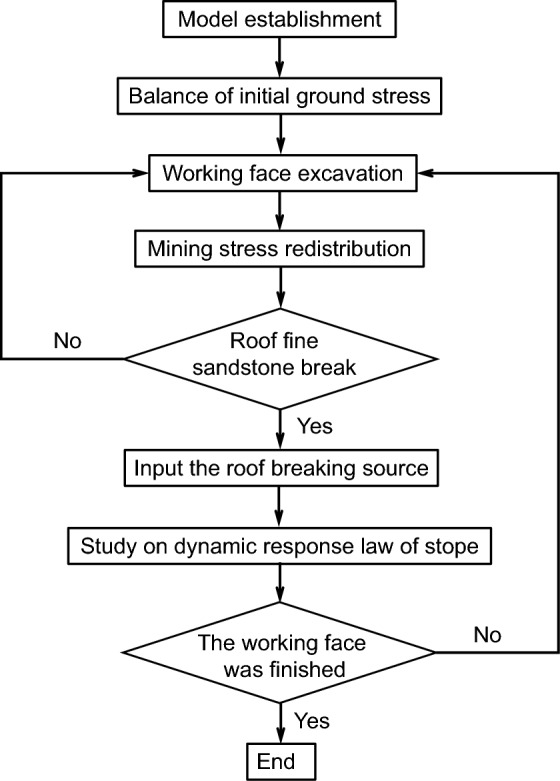
Flow chart.

To analyze the dynamic response of the roadway induced by the breaking of the hard roof during the working face extraction, the numerical computation can be divided into two stages: static calculation and dynamic calculation. The numerical computation process is illustrated in Fig. 6.

The initial geostress balance, roadway excavation, and working face extraction are all conducted during the static analysis stage. The primary purpose of static calculation is to obtain the stress distribution of the stope during the excavation of the working face. When the working face advances to a certain step, the roof will experience first and periodic breaking. Once the roof of fine sandstone breaks and releases energy, we initiate the dynamic analysis module. The mining source released by hard roof breaking is input into the calculation model in the form of seismic moment *M*_*0*_. The magnitude of seismic moment *M*_*0*_ is calculated based on Eq. 6^25,26^, and the energy released *E* from the roof breaking obtained from theoretical analysis in Chapter 3. The simulation of the source induced by hard roof breaking provides a basis for us to more accurately study the dynamic response characteristics of roadway. It is important to note that during dynamic analysis, the boundary conditions of the model are changed from fixed boundary conditions to static boundaries to eliminate refraction and reflection of dynamic stress waves at the model boundaries^27^. The research shows that using local damping to simulate the dynamic analysis of rock mechanics can achieve ideal results, and the local damping of rock is generally 2–5%, so the local damping is selected as 5% in the dynamic calculation^28^.

6 $$E=\frac{{M}_{0}}{2\times {10}^{4}}$$

### Ethical approval

This article does not contain any studies with human participants or animals performed by any of the authors.

## Simulation results and analysis

### Vibration velocity of the roadway under dynamic disturbance from roof breaking

The breaking of hard roof releases a large amount of energy, which will cause the vibration of coal and rock mass near the working face. There is a significant correlation between the vibration velocity and the damage of the rock mass^29,30^. When the peak particle velocity (PPV) of the rock mass is high, it can even induce severe deformation of the roadway and even rockburst accidents. Therefore, analyzing the evolution law of vibration velocity of surrounding rock of roadway under the condition of hard roof breaking is of great significance for evaluating the stability of the roadway.

**Figure 7:**
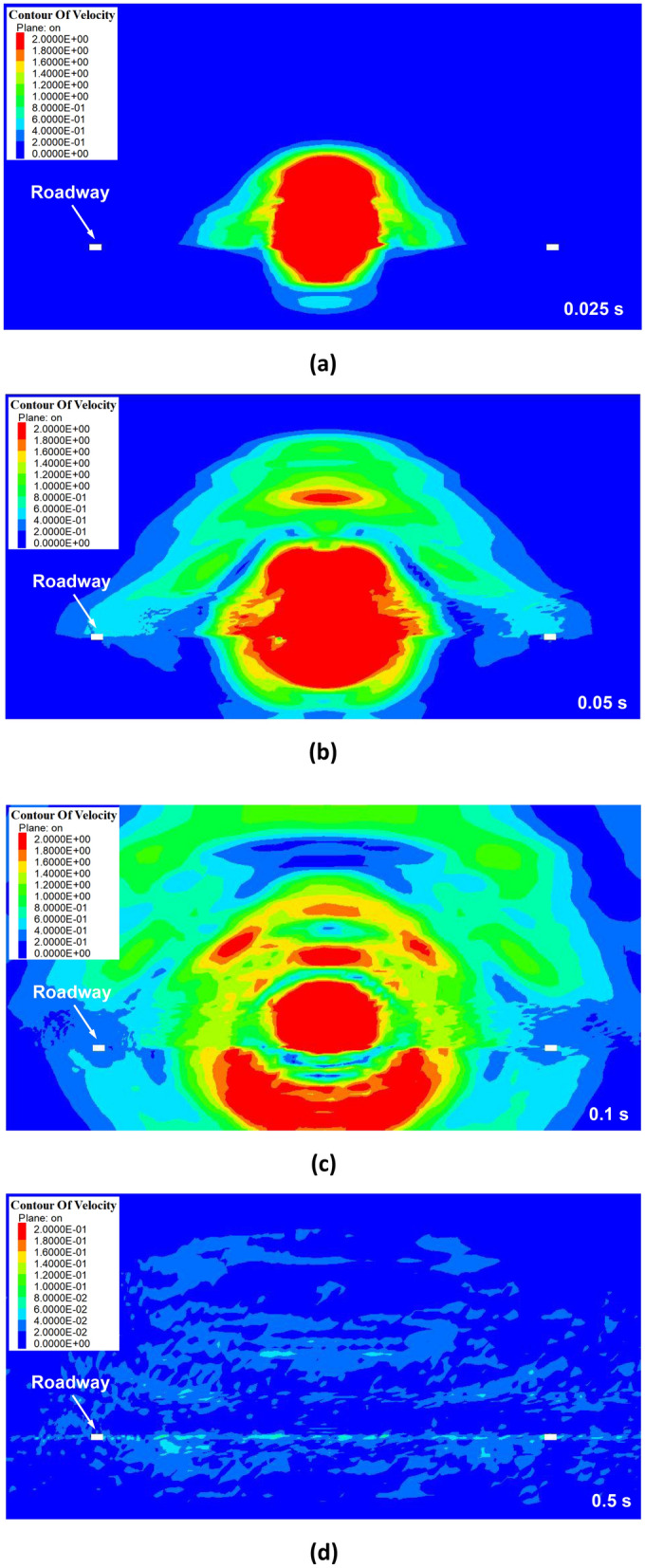
Cloud map of stope vibration velocity during the first weighting (**a**) the dynamic disturbance last for 0.025 s (**b**) the dynamic disturbance last for 0.05 s (**c**) the dynamic disturbance last for 0.1 s (**d**) the dynamic disturbance last for 0.5 s.

Figure 7 depicts a cloud map of vibration velocity propagation in the stope when the hard roof first weighting. From the diagram, it is evident that the vibration velocity at the source is the largest, after that, the vibration continued to spread around. As the vibration propagates towards to the roadway, it potentially triggers dynamic hazards such as roadway instability or rockburst. During the propagation of the vibration waves, there is continuous attenuation in vibration velocity magnitude. After 0.5 s of dynamic disturbance, there is a noticeable decline of the vibration velocity, dropping below 0.2 m/s. After 0.5 s, the impact of hard roof breaking on the surrounding rock of the mining area is significantly reduced.

**Figure 8:**
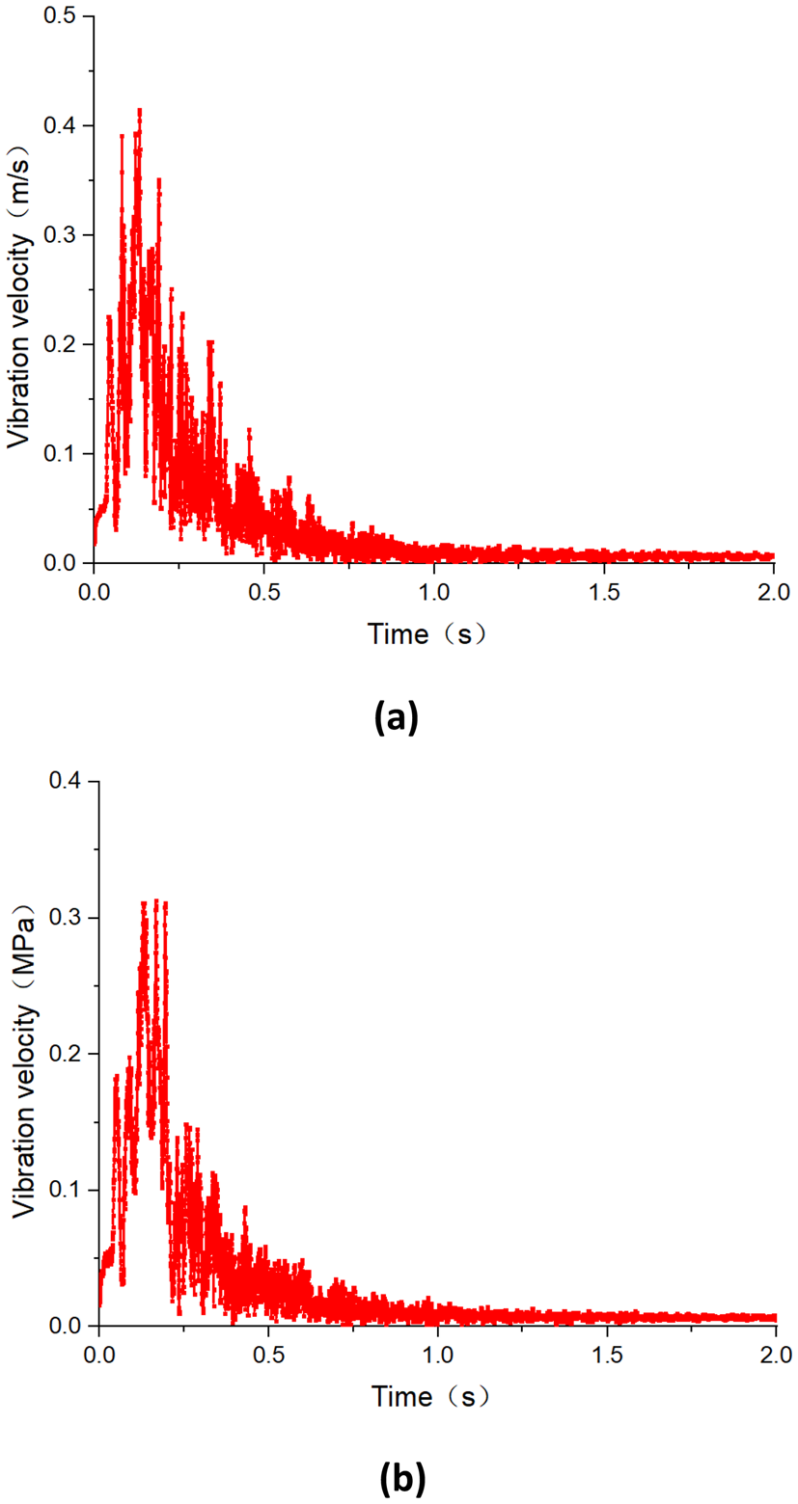
Vibration velocity of the roadway surrounding rock during the first weighting (**a**) right side of the roadway (**b**) left side of the roadway.

**Figure 9:**
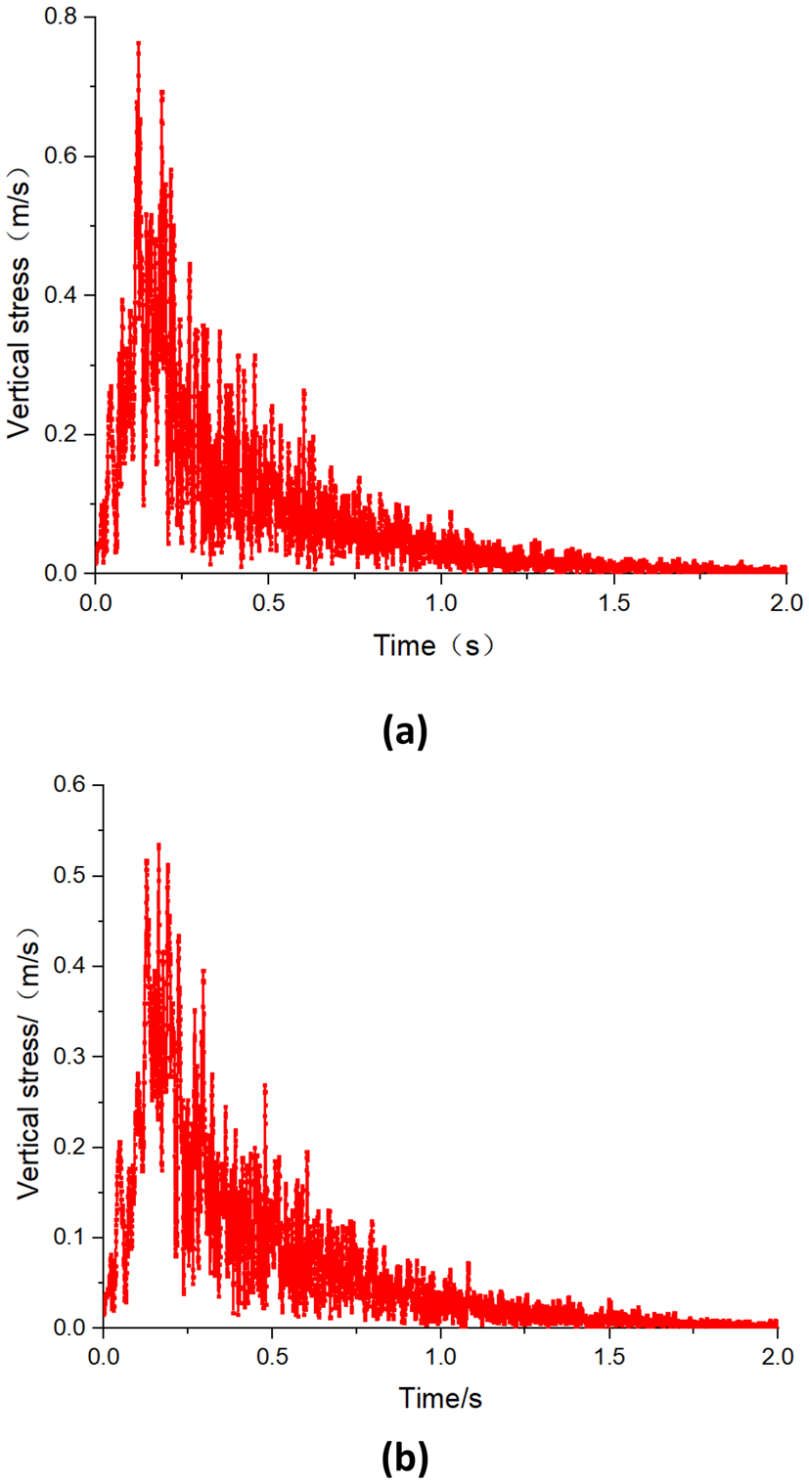
Vibration velocity of the roadway surrounding rock during the periodic weighting (**a**) right side of the roadway (**b**) left side of the roadway.

Figures 8 and 9 depict the vibration velocity curves of roadway during the first weighting and periodic weighting, respectively. From the graphs, it is observed that during the first weighting, the maximum vibration velocities on the left and right sides of the roadway are 0.42 m/s and 0.32 m/s, respectively. Whereas, during the periodic weighting, the PPV on the right and left sides of the roadway are 0.78 m/s and 0.54 m/s, respectively. The PPV on the right side of the roadway (mining side of the roadway) is greater than that on the left side. Furthermore, during the periodic weighting, both the PPV and the duration of vibration in the roadway are greater than those during the first weighting. When the duration of dynamic disturbance during the first weighting is less than 0.5 s, the vibration velocity of the roadway surrounding rock is relatively high, remaining above 0.05 m/s. Similarly, during the periodic weighting, when the duration of dynamic disturbance is less than 1.0 s, the vibration velocity generally remains above 0.05 m/s.

### Dynamic response of the stress in the roadway

#### Dynamic response of the stress in the roadway during the first weighting of the hard roof

**Figure 10:**
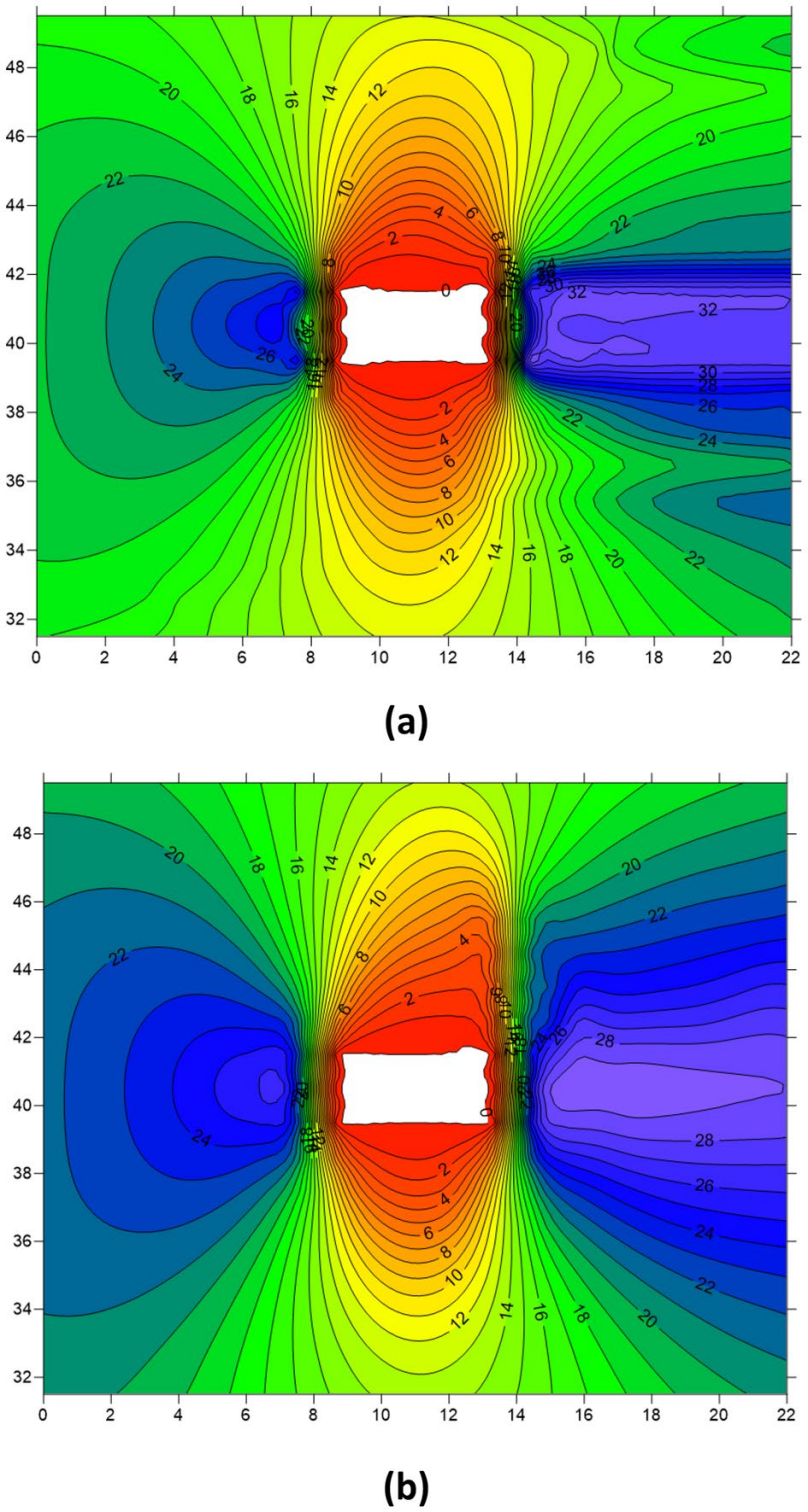
Cloud map of stress in roadway before and after the hard roof first weighting (**a**) Before the first weighting (**b**) After the first weighting.

Figure 10 presents the stress distribution profiles of the roadway 5 m ahead of the working face. Figure 10a illustrates the stress distribution of the roadway before the first weighting of the roof, while Fig. 10b shows the stress distribution after the first weighting. From the figures, it is evident that excavation of the working face leads to noticeable stress concentration on both sides of the roadway, with a greater degree and extent of stress concentration observed on the right side (mining side of the roadway) compared to the left side. After the dynamic disturbance caused by the breaking of the hard roof, there is a redistribution of stress in the roadway surrounding rock. Although there is a slight decrease in the stress peak values on both sides of the roadway, there is a significant increase in the extent of stress concentration, particularly noticeable on the right side. Thus, dynamic disturbance can expand the influence range of mining-induced stress to a certain extent.

**Figure 11:**
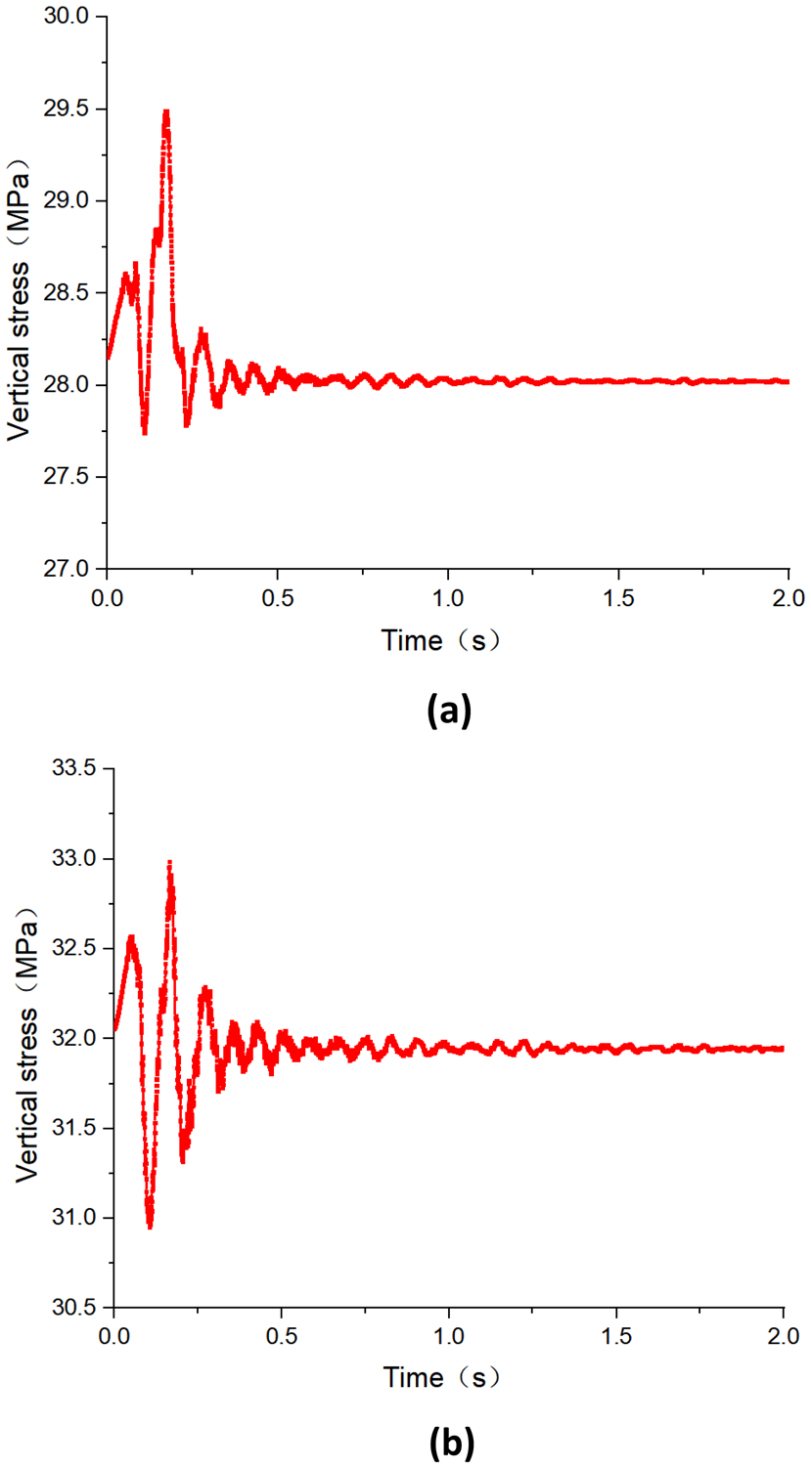
The variation pattern of peak vertical stress of the roadway under the dynamic disturbance of first weighting (**a**) Left side of the roadway (**b**) Right side of the roadway.

Figure 11 depicts the variation of peak vertical stress on both sides of the roadway under dynamic disturbance caused by hard roof breaking. Figure 11a illustrates the variation of peak stress on the left side of the roadway, while Fig. 11b shows the variation on the right side. It can be observed from the figure that under the first weighting dynamic disturbance, there is a significant oscillation in the roadway surrounding rock stress, especially within the first 0.5 s after roof breaking, during which the stress oscillation is intense. Beyond 0.5 s, the amplitude of stress oscillation notably decreases, after 1 s the disturbance caused by dynamic load gradually subsides, and the stress no longer shows significant changes. Before the dynamic disturbance caused by roof breaking, the peak stress on the right side of the roadway was 32.1 MPa. After the dynamic disturbance, it decreased to 31.9 MPa, indicating a decrease of 0.2 MPa. Similarly, before the dynamic disturbance perturbation, the peak stress on the left side of the roadway was 28.2 MPa, which decreased to 28.0 MPa after the dynamic disturbance, also decrease 0.2 MPa. Combining with the variation trend of vibration velocity in roadway as discussed in section "Dynamic analysis procedures", it can be inferred that the magnitude of vibration velocity in roadway corresponds to the amplitude of stress oscillation. That is, the greater the vibration velocity, the greater the amplitude of stress oscillation.

#### Dynamic response of the stress in the roadway during the periodic weighting of the hard roof

**Figure 12:**
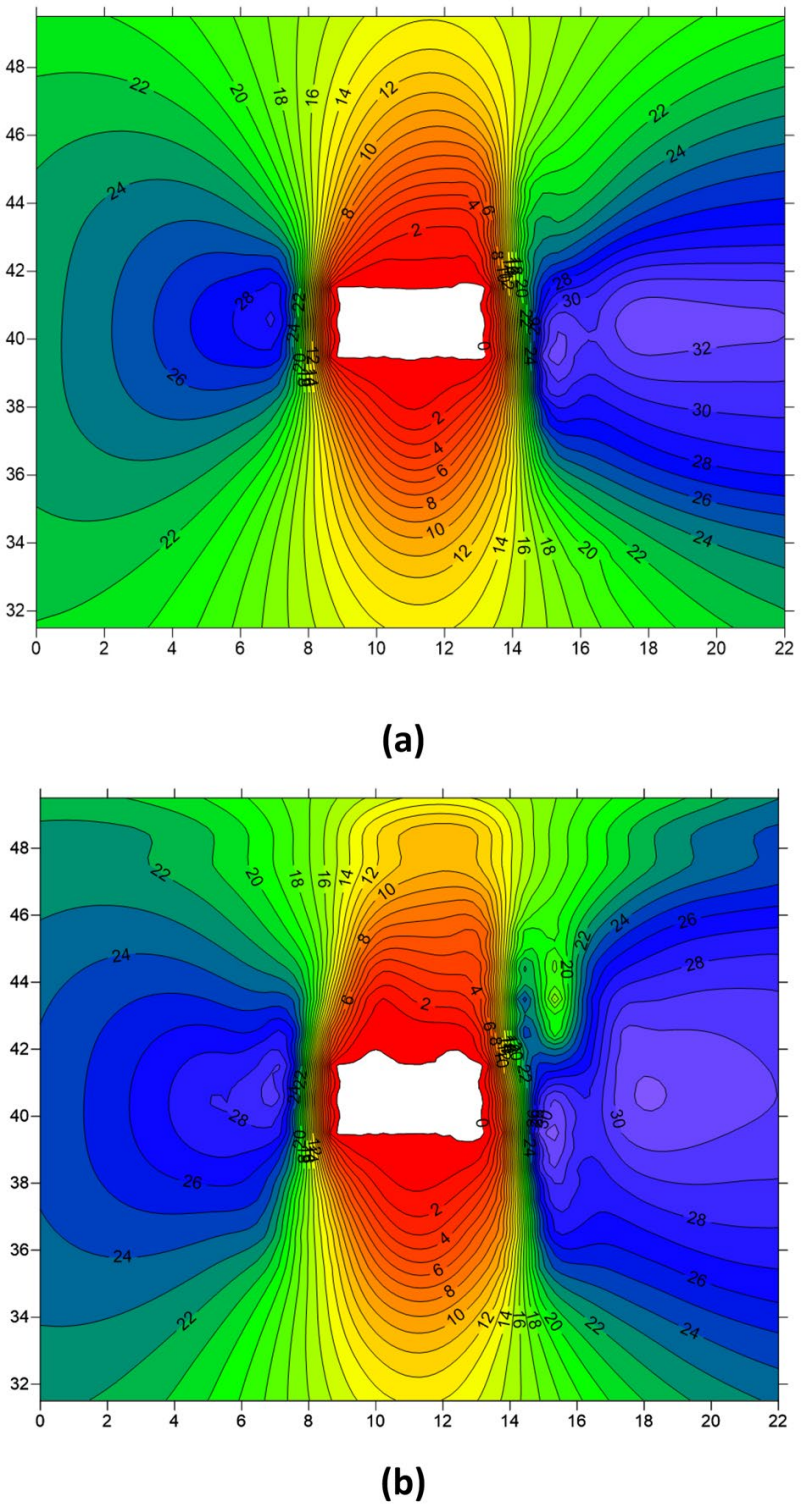
Cloud map of roadway surrounding rock stress before and after the period weighting of the hard roof (**a**) Before periodic weighting (**b**) After periodic weighting.

Figure 12a illustrates the stress distribution of the roadway before periodic weighting of the roof, while Fig. 12b depicts the stress distribution after the periodic weighting following the dynamic disturbance caused by hard roof breaking. From the figures, it can be observed that compared to the first weighting, the stress concentration in the roadway surrounding rock is higher during periodic weighting due to the larger extent of the goaf. Additionally, following the dynamic disturbance caused by the breaking of the hard roof, there is a decrease in stress on both sides of the roadway, accompanied by a noticeable expansion in the concentration range of stress along the sidewalls. Moreover, the variation of stress is more pronounced on the right side of the roadway, indicating a more significant disturbance experienced by the right sidewall due to the breaking of the hard roof.

**Figure 13:**
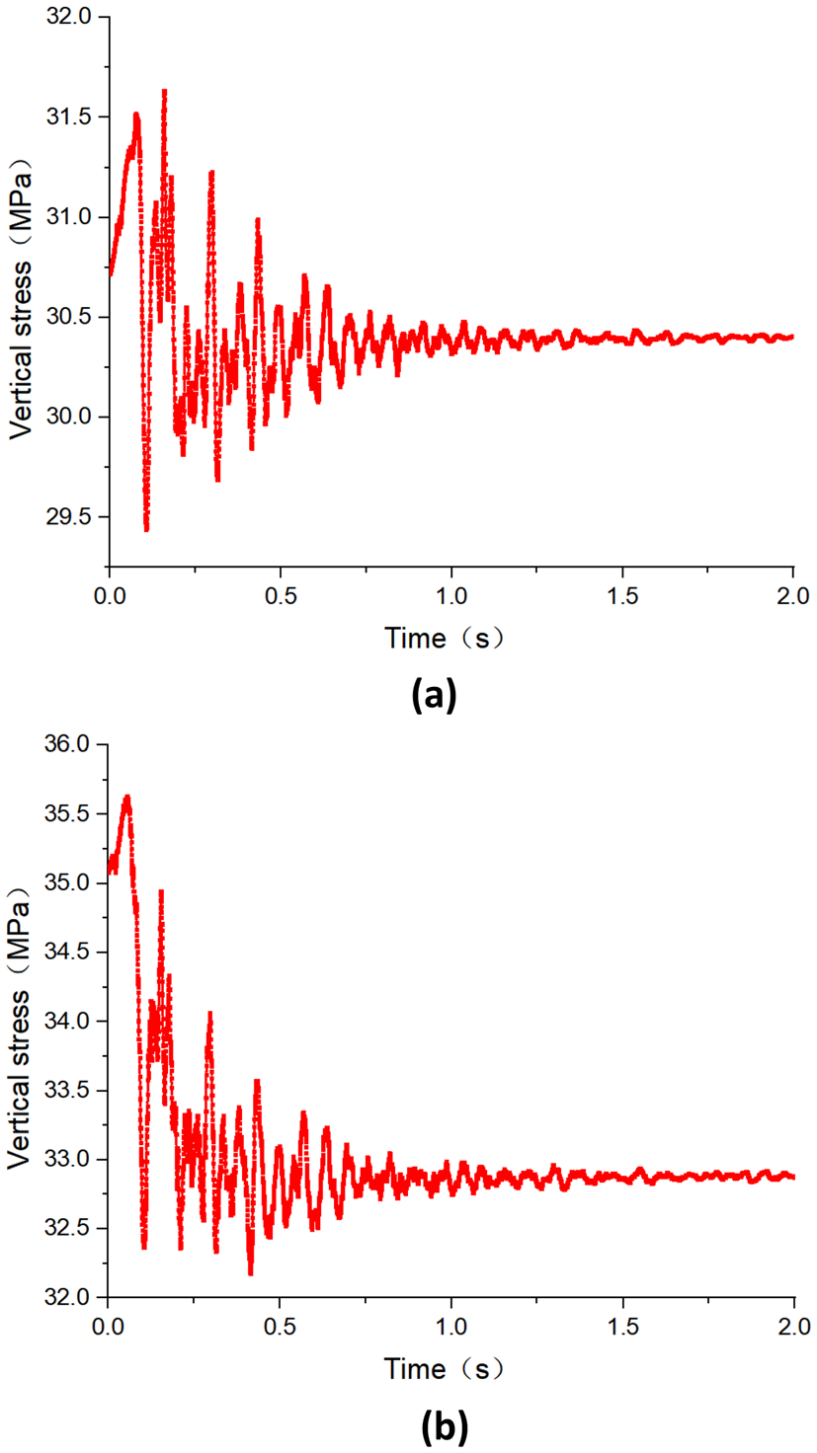
The variation pattern of peak vertical stress of the roadway under the dynamic disturbance of periodic weighting (**a**) Left side of the roadway (**b**) Right side of the roadway.

Figure 13 illustrates the variation patterns of peak vertical stress on both sides of the roadway under dynamic disturbances caused by roof periodic breaking. Specifically, Fig. 13a represents the variation of peak stress on the left side of the roadway, while Fig. 13b depicts the variation on the right side. Similar to the first weighting of hard roof, the periodic weighting also induces significant oscillations of stress in the roadway surrounding rock, although the duration of intense oscillations is noticeably longer than during the first weighting. Within the first 0.75 s after periodic weighting, stress oscillations are intense, followed by a notable decrease in amplitude after 0.75 s. The dynamic disturbances gradually diminish after 1.5 s, with stress gradually stabilizing. Before the dynamic disturbances caused by periodic weighting, the peak stress on the right side of the roadway was 35.1 MPa, which decreased to 32.9 MPa afterward, indicating a decrease of 2.2 MPa. Similarly, the peak stress on the left side of the roadway decreased from 30.7 MPa before dynamic disturbance to 30.4 MPa afterward, reflecting a decrease of 0.3 MPa. The reduction in peak stress on the roadway under dynamic disturbances caused by periodic weighting exceeds that during the first weighting, with the reduction in stress being notably greater on the right side compared to the left. Compared to the first weighting, the reduction of stress during periodic weighting is greater, partly due to the release of more energy during periodic weighting and partly because the static stress level of the roadway surrounding rock is higher, making it more sensitive to dynamic disturbance.

### The dynamic response characteristics of the plastic zone of the roadway

**Figure 14:**
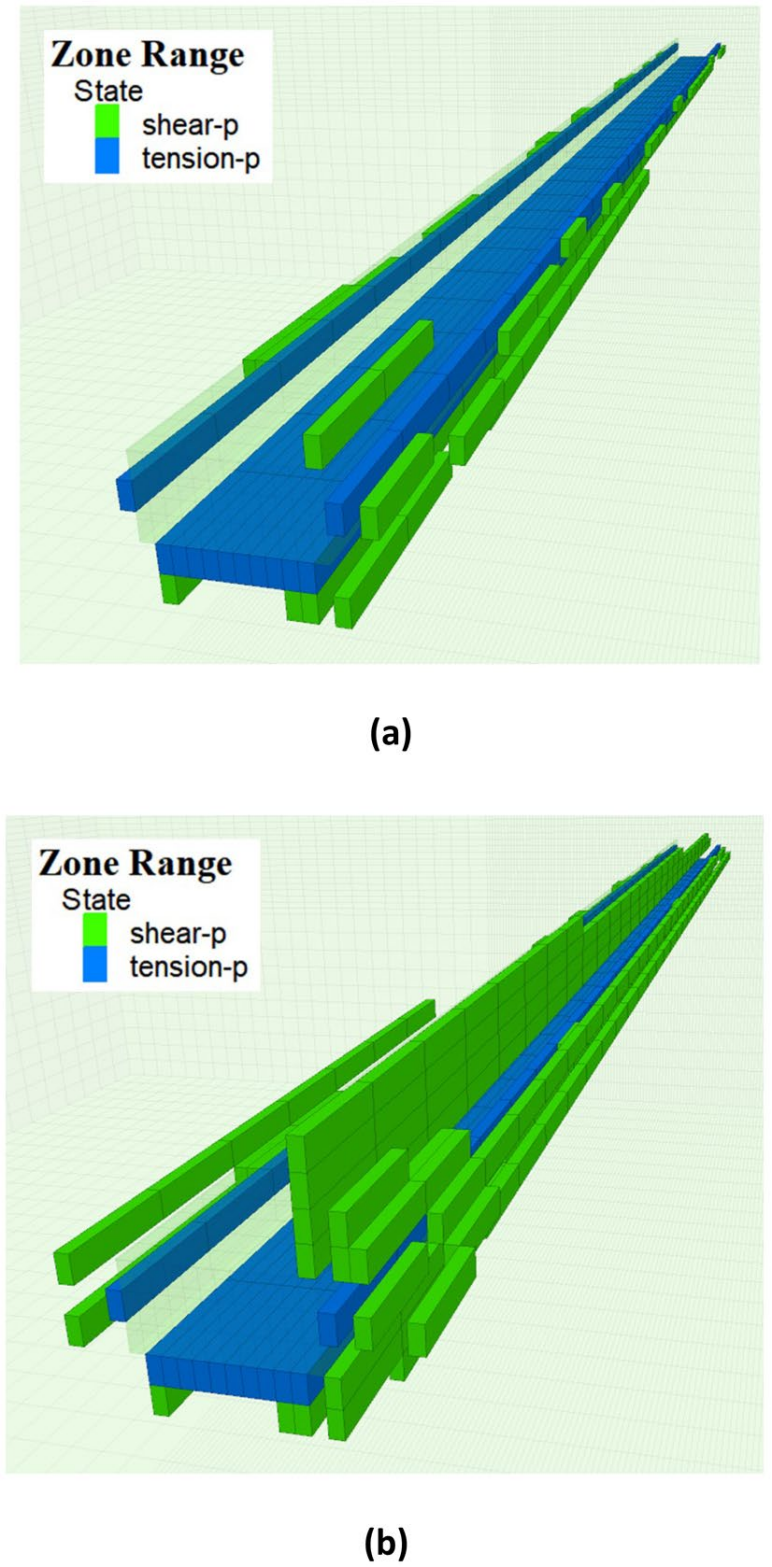
The development of plastic zones of the roadway before and after the first weighting (**a**) Before the first weighting (**b**) After the first weighting.

**Figure 15:**
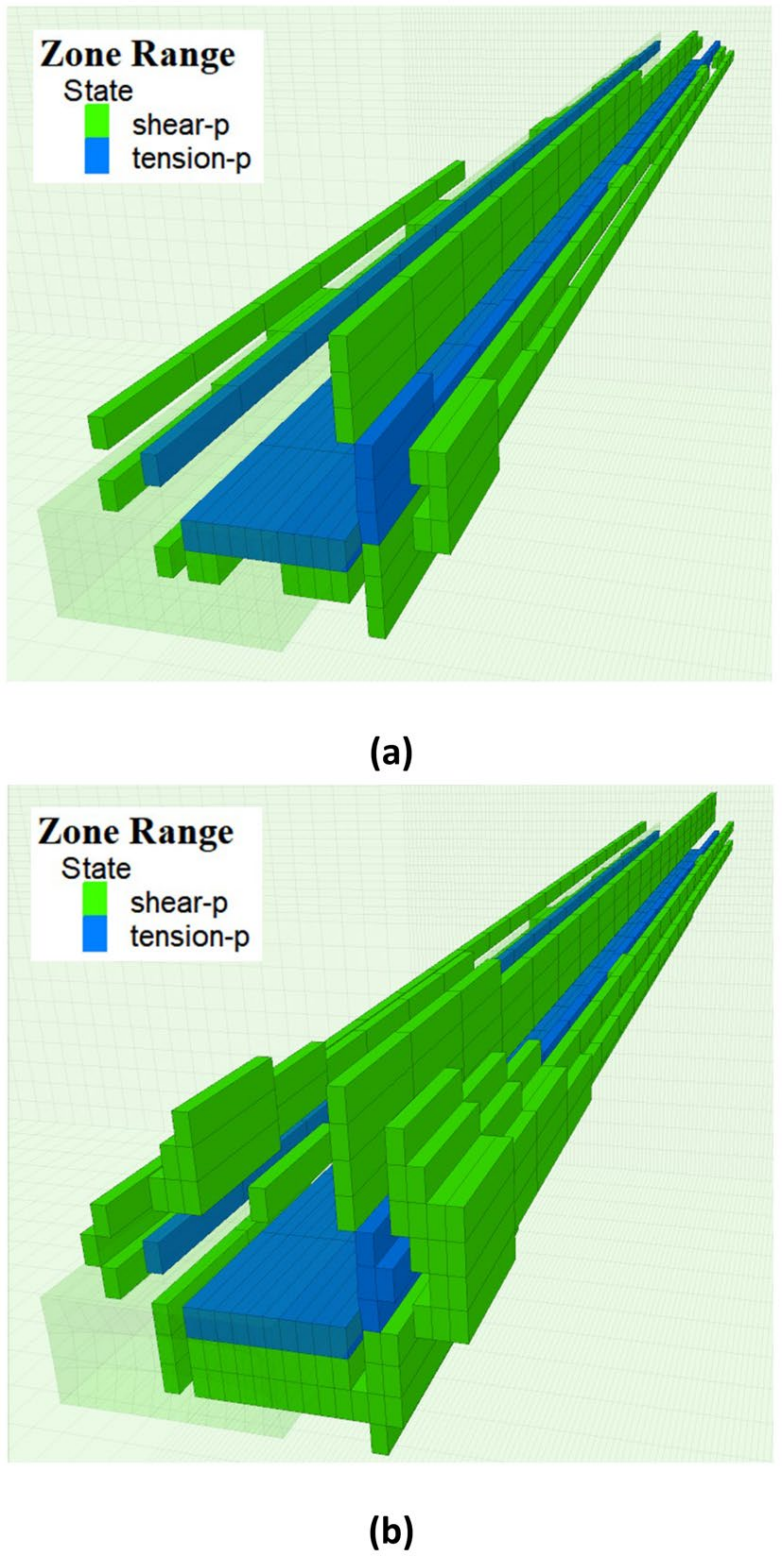
The development of plastic zones of the roadway before and after periodic weighting (**a**) Before periodic weighting (**b**) After periodic weighting.

Figures 14 and 15 illustrate the distribution patterns of the plastic zones of the roadway before and after the first and periodic weighting. It is evident from the figures that under the dynamic disturbance of roof breaking, the plastic zones of the roadway's roof, floor, and sidewalls expand. Before the first weighting, the development widths of the plastic zones in the roadway's roof, floor, right sidewall and left sidewall were 1 m, 2 m, 1.5 m and 0.5 m, respectively. After the dynamic disturbance of the first weighting, these widths increased to 4 m, 2 m, 3 m and 2 m, respectively. Before the periodic weighting, the development widths of the plastic zones in the roadway's roof, floor, right sidewall and left sidewall were 3 m, 3 m, 3.5 m and 2.5 m, respectively. After the dynamic disturbance of the periodic weighting, these widths expanded to 4 m, 4 m, 4 m and 2.5 m, respectively. During the hard roof breaking, the stress of the roadway surrounding rock reaches the strength of the coal-rock mass under the combined effect of dynamic and static loads, resulting in plastic deformation of the coal-rock mass. Consequently, the peak static stress decreases, and the stress peak transfers deeper into the surrounding rock of the roadway, leading to the influence range of mining induced stress increases.

### The dynamic response patterns of roadway deformation

**Figure 16:**
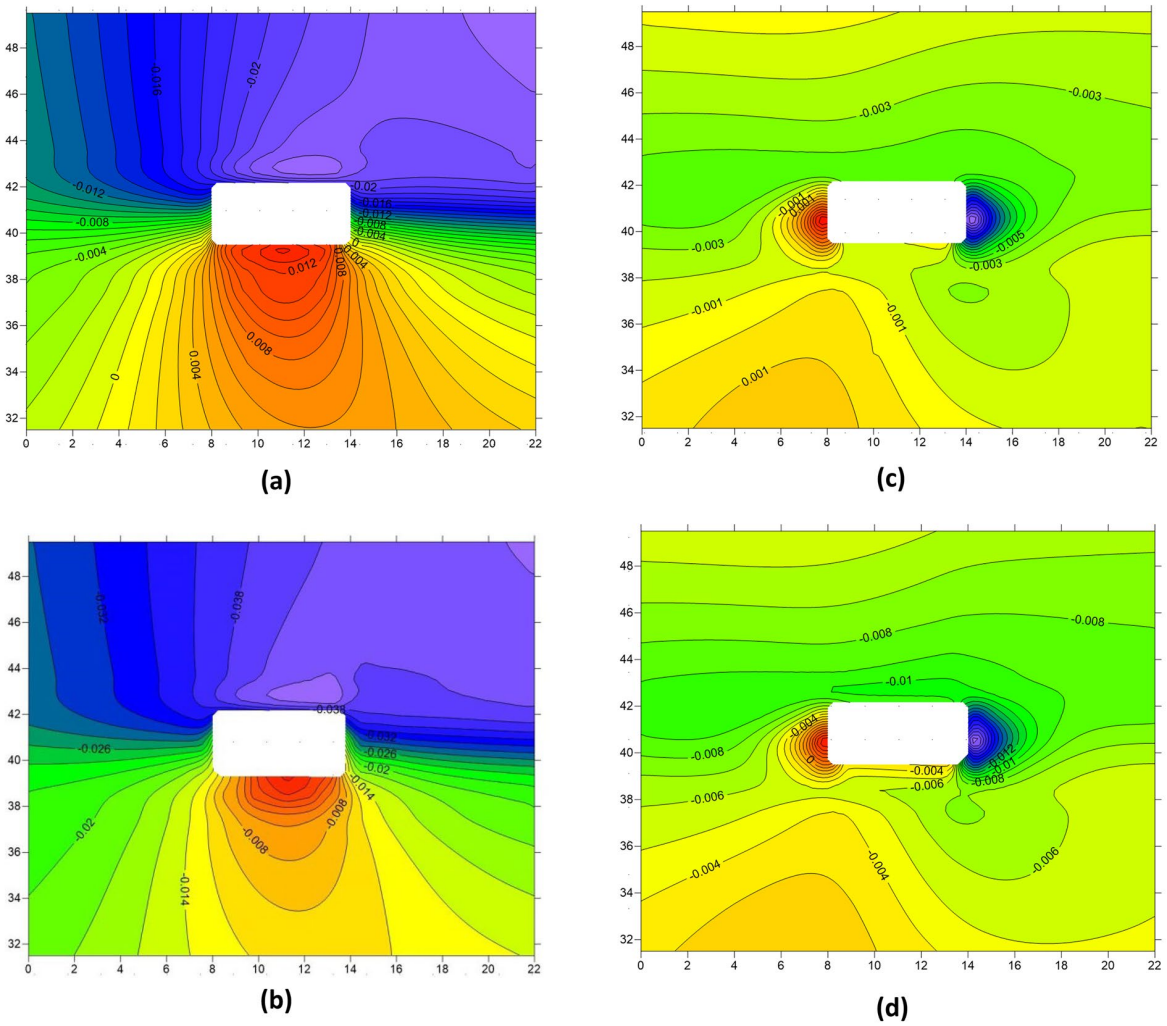
Deformation of the roadway before and after the first weighting (**a**) Vertical displacement of the roadway before first weighting (**b**) Vertical displacement of the roadway after first weighting (**c**) Horizontal displacement of the roadway before first weighting (**d**) Horizontal displacement of the roadway after first weighting.

**Figure 17:**
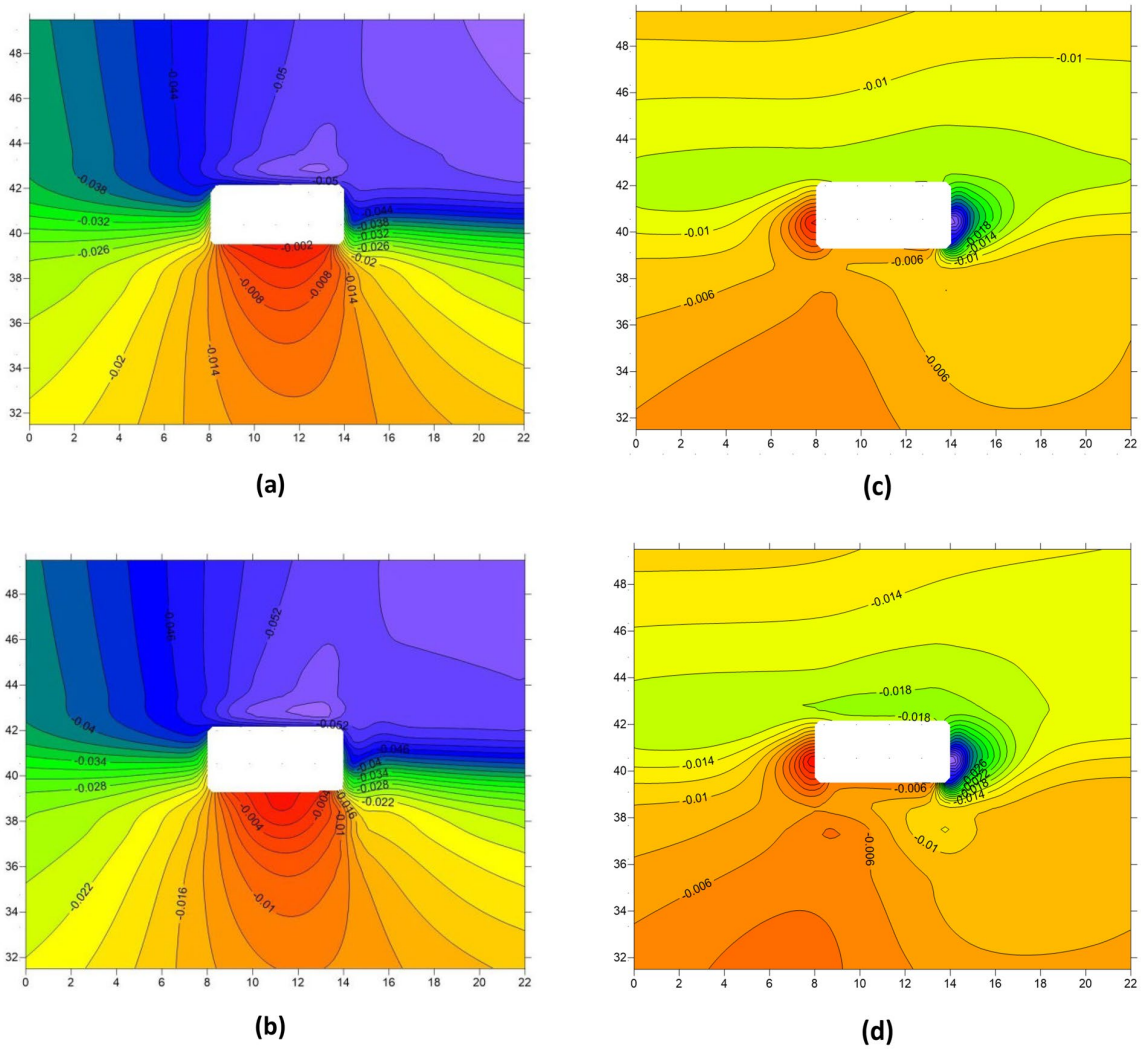
Deformation of the roadway before and after the periodic weighting (**a**) Vertical displacement of the roadway before periodic weighting (**b**) Vertical displacement of the roadway after periodic weighting (**c**) Horizontal displacement of the roadway before periodic weighting (**d**) Horizontal displacement of the roadway after periodic weighting.

Figures 16 and 17, along with Table 2, present the deformation patterns of the roadway before and after the first and periodic weighting. By combining the figures and the table, it is evident that the deformation patterns of the roadway are significantly affected by hard roof breaking, with the roadway roof and left sidewall being particularly influenced by the dynamic disturbance caused by hard roof breaking. During the first weighting, the maximum deformation of the roadway roof increased from 0.023 to 0.043 m, with a growth of 0.02 m and a growth rate of 87.0%. During the periodic weighting, the maximum deformation of the roadway left sidewall increased from 0.041 to 0.057 m, with an increase of 0.016 m and a growth rate of 39.0%. Apart from the roadway roof and left sidewall, the roadway floor and right sidewall also experienced varying degrees of changes due to the effect of hard roof breaking, with an overall trend of increased deformation.

**Table 2:** The deformation patterns of the roadway under dynamic disturbance disturbances caused by hard roof breaking.

	Deformation amount(m)				Variation amount(m)			
	Roof	Floor	Left sidewall	Right sidewall	Roof	Floor	Left sidewall	Right sidewall
Before first weighting	0.023	0.013	0.011	0.019	+ 0.02	− 0.007	− 0.004	+ 0.005
After first weighting	0.043	0.005	0.007	0.024				
Before period weighting	0.053	0.006	0.007	0.041	+ 0.003	+ 0.002	+ 0.002	+ 0.016
After period weighting	0.056	0.008	0.009	0.057				

## Stability assessment of roadway

The primary reason for severe deformation or even rockburst of roadway is the high concentration of stress. The stress of roadway surrounding rock can be divided into two components: static stress and dynamic disturbance stress. Based on stress superposition theory, the total stress *σ* of roadway surrounding rock can be expressed as the sum of static stress *σ*_*s*_ and dynamic disturbance stress *σ*_*d*_. Using the ratio of total stress of roadway surrounding rock to its uniaxial compressive strength *σ*_c_ as an index *I*_*c*_ for assessing roadway stability, the level of dynamic disaster risk in roadways can be classified^31,32^.

The dynamic stress *σ*_*d*_ generated by roof fracture can be expressed as:

7 $${\sigma }_{d}=\rho C{v}_{0}$$

In the formula *ρ* represents the density of the coal mass, *C* is the propagation velocity of the wave, and *v*_0_ is the maximum vibration velocity of the roadway surrounding rock.

The stability index *I*_*c*_ of the roadway can be expressed as:

8 $${I}_{c}=\frac{{\sigma }_{s}+\rho C{v}_{0}}{\left[{\sigma }_{c}\right]}$$

Furthermore, based on the magnitude of the roadway stability discrimination index *I*_*c*_, it is classified into four levels: safe, slight risk, moderate risk, and severe risk. The classification of roadway stability is shown in Table 3.

**Table 3:** Classification of roadway stability.

0–1.5	1.5–2.0	2.0–2.5	> 2.5
Safe	Slight risk	Moderate risk	Severe risk

**Figure 18:**
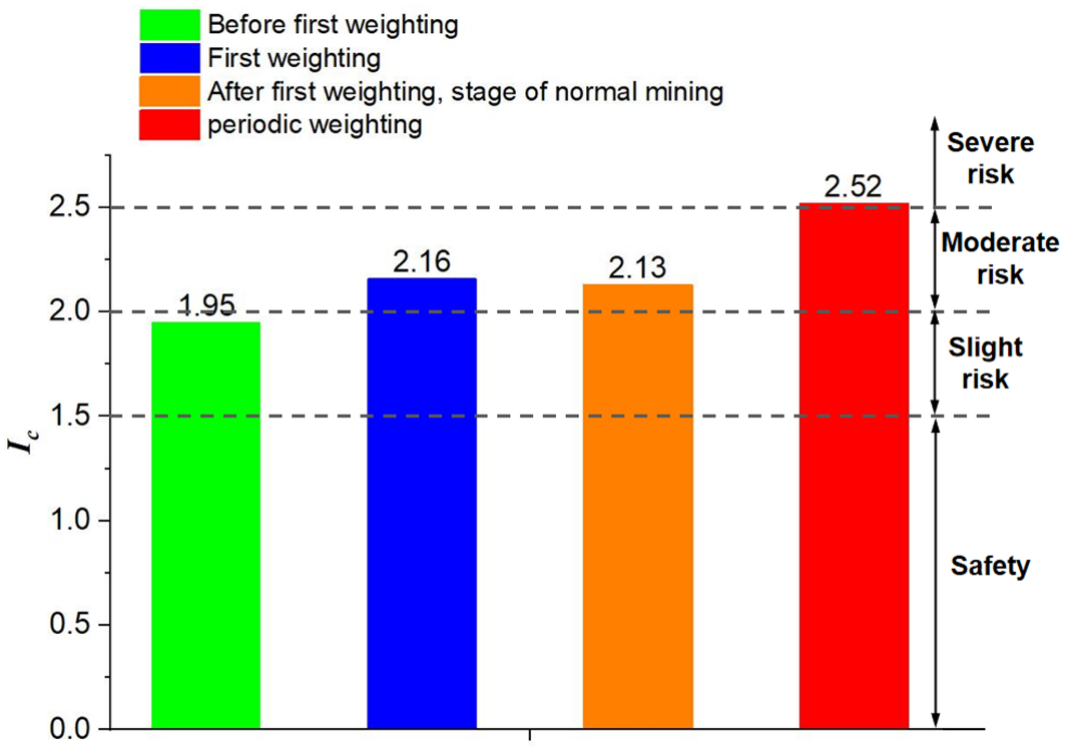
Assessment of roadway stability.

Based on the roadway stability discrimination index *I*_*c*_ and the classification of roadway stability, an assessment of roadway stability during the working face excavation is conducted through numerical simulation calculations. In the calculation process of the roadway stability discrimination index *I*_*c*_, the propagation velocity of waves is taken as 5600 m/s, and the density and compressive strength of the coal mass are taken as 1480 kg/m^3^ and 16.5 MPa, based on experimental test results. The roadway stability index and danger level during the working face excavation period are shown in Fig. 18. It can be observed from the figure that during the working face excavation period, the roadway stability discrimination index *I*_*c*_ is 1.95 before the first weighting, 2.16 during the first weighting, 2.13 after the first weighting during normal excavation, and 2.53 during periodic weighting, with corresponding roadway stability levels of slight danger, moderate danger, moderate danger, and severe danger, respectively.

The roadway stability evaluation index *I*_c_ proposed by consider the static stress of roadway and the dynamic load disturbance of the hard roof breaking. Therefore, compared with the traditional evaluation index that only consider static stress, the roadway stability index *I*_c_ calculated during the hard roof breaking period is larger, indicating that the roadway is more likely to be instability during the roof breaking period. The calculation result of the roadway stability evaluation index *I*_*c*_ is more consistent with the actual production situation, indicating that the evaluation index can more accurately evaluate the roadway stability under the condition of hard roof.

## Conclusion

The article employs a comprehensive approach integrating theoretical analysis and numerical simulation methods to investigate the breaking behavior and energy release patterns of the hard roof directly overlying working face. It proposes a dynamic calculation method for hard roof break and explores the dynamic response characteristics of roadway surrounding rock. Furthermore, it introduces evaluation indicators for roadway stability under the disturbance of hard roof breaking, thereby providing a theoretical basis for controlling roadway stability in hard roof environments. The primary conclusions of the research are summarized as follows.

1. Based on thin plate theory and mining pressure theory, the study investigated the break patterns and the magnitude of energy release of the hard roof directly overlaying the 11,618(east)working face, providing a theoretical basis for predicting roof breaking. For the hard roof of fine sandstone, the first breaking step distance is 43.31 m, releasing the elastic energy of 5.0 × 10^6^ J. The step distance of periodic breaking was 43.29 m, releasing the elastic energy of 8.2 × 10^6^ J. The elastic energy released during periodic break was higher than that released during the first break.
2. Based on the dynamic analysis module of FLAC3D numerical simulation software, a dynamic calculation simulation method for hard roof breaking was proposed. This method enabled the comprehensive simulation of the entire process of "working face excavation—hard roof breaking—dynamic response of roadway." The study investigated the dynamic response characteristics of roadway surrounding rock under the disturbance of hard roof break, including vibration velocity, stress, displacement, etc. The results indicate that after the disturbance of hard roof breaking, there was a slight decrease in peak stress on both sides of the roadway, accompanied by a significant increase in the range of stress concentration and expansion of the plastic zone. Indicating that dynamic disturbance can expand the influence range of mining-induced stress to a certain extent. Roadway deformation patterns were severely affected by roof breaking, with the roadway roof and the right side (mining side of the roadway) being the most significantly influenced by the dynamic impact of roof breaking.
3. Considering the dynamic disturbance effects of hard roof breaking, evaluation criteria and stability classification levels for roadway under the disturbance of hard roof break were introduced. An assessment of roadway stability during the working face excavation was conducted. Before the first weighting, during the first weighting, after the first weighting during normal excavation, during periodic weighting, the roadway stability levels were categorized as slight risk, moderate risk, moderate risk, and severe risk, respectively. During the period of hard roof breaking, the possibility of instability of the roadway increased significantly due to the disturbance caused by the dynamic load induced by the hard roof breaking.

## Data Availability

The datasets generated during and/or analysed during the current study are available from the corresponding author on reasonable request.
